# Case Report: A leadless and endovascular pacemaker teamwork

**DOI:** 10.3389/fcvm.2023.1287506

**Published:** 2023-11-09

**Authors:** Sarah Zeriouh, Vasileios Sousonis, Roberto Menè, Serge Boveda, Quentin Voglimacci-Stephanopoli, Stéphane Combes

**Affiliations:** Heart Rhythm Management Department, Clinique Pasteur, Toulouse, France

**Keywords:** leadless pacemaker, endovascular pacemaker, atrioventricular synchrony, lead dysfunction, device infection

## Abstract

**Background:**

Cardiac Implantable Electronic Device infections increase short- and long-term mortality, along with healthcare costs. Leadless pacemakers (PM) were developed to overcome pocket- and minimize lead-related complications in selected high-risk patients. Recent advancements enable leadless devices to mechanically detect atrial activity, facilitating atrioventricular (AV) synchronous stimulation.

**Case summary:**

A 90-year-old woman, implanted with a dual-chamber pacemaker eight years ago due to sinus node dysfunction, presented with syncope. A diagnosis of complete AV block, in the setting of ventricular lead dysfunction was made. Due to a high risk of infection, the patient was implanted with a leadless PM capable of maintaining AV synchrony in VDD mode (MICRA™ model MC1AVR1). The transvenous PM was programmed to AAI-R mode to drive the atria, which, in turn, triggered the leadless PM to stimulate the ventricles. At six month follow-up, the AV synchrony rate was 85%.

**Conclusion:**

The combination of classic atrial pacing with leadless ventricular stimulation can be used in high-risk patients to reduce the risk of complications, in the setting of ventricular lead dysfunction. In this manner, AV synchrony can be maintained, improving hemodynamic parameters and quality of life. Low sinus rate variability at rest is essential to achieve a high AV synchrony rate in such cases.

## 1. Introduction

Cardiac Implantable Electronic Devices (CIEDs) reduce morbidity and mortality in appropriately selected patients. These benefits may be mitigated by complications such as infections, that increase short- and long-term mortality along with healthcare costs (1). Several risk factors for CIED-related infections have been identified and include: age, the lack of antibiotic prophylaxis, diabetes, renal impairment, the use of corticosteroids and early reinterventions (2). Leadless pacemakers (PM) were developed to overcome pocket- and minimize lead-related complications. In order to preserve atrioventricular (AV) synchrony, recent models are equipped with an accelerometer-based atrial sensing algorithm. In this case report, we present a patient at high risk of infection, with complete AV block due to dysfunction of a ventricular pacing lead, in whom AV synchrony was achieved with the combination of a MICRA AV and a transvenous dual-chamber PM.

## 2. Case description

**Figure 1:**
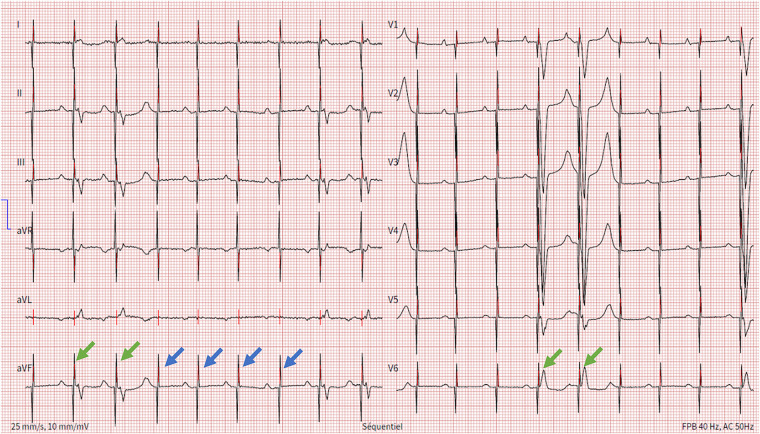
Electrocardiogram showing spontaneous P wave activity followed by unipolar ventricular stimulation spikes that most of the times are ineffective (blue arrows) and rarely result in ventricular capture (green arrows).

A 90-year-old woman presented to the Emergency Department for syncope. Her past medical history included a moderate chronic kidney disease and the implantation of a dual-chamber PM for symptomatic sinus node dysfunction eight years prior to her presentation. Of note, PM implantation was complicated by an early displacement of the ventricular lead, necessitating a reintervention on the first day following the procedure. At her admission, the patient was confused and hypotensive with a blood pressure of 89/56 mmHg and a heart rate of 35 beats per minute. Physical examination revealed no signs of heart failure or heart murmurs. Laboratory results showed a potassium level of 5,6 mmol/L (normal ranges 3,5–5,0 mmol/L), a glomerular filtration rate of 38 ml/min and a C-reactive protein of 7,6 mg/L (normal ranges < 5 mg/L). The electrocardiogram revealed a complete AV block due to intermittent loss of capture of the ventricular lead (Figure 1). Echocardiography showed a preserved right and left ventricular systolic function, without significant heart valve disease. The interrogation of the PM revealed an elevation of the ventricular stimulation threshold (3,5 V at a pulse width of 0,5 ms), compared with 0,7 V at a pulse width of 0,5 ms three months ago, along with a ventricular sensing at 8 mV and a stable impedance of 480 ohms. Corresponding parameters for the atrial lead included a stimulation threshold of 0,6 Volts at a pulse width of 0,5 ms, a sensing of 0,4 mV and an impedance of 420 ohms. Battery's longevity was estimated at three years. Chest x-ray did not show any macroscopic lead fracture or displacement. Based on the above, the diagnosis of a syncope due to complete AV block in the setting of ventricular lead dysfunction was made.

**Figure 2:**
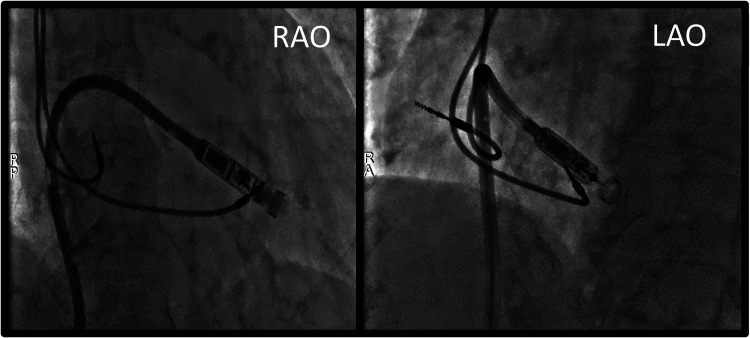
Procedural fluoroscopy images showing leadless PM's position at the level of the lower interventricular septum, just before device deployment. RAO, right anterior oblique; LAO, left anterior oblique.

**Figure 3:**
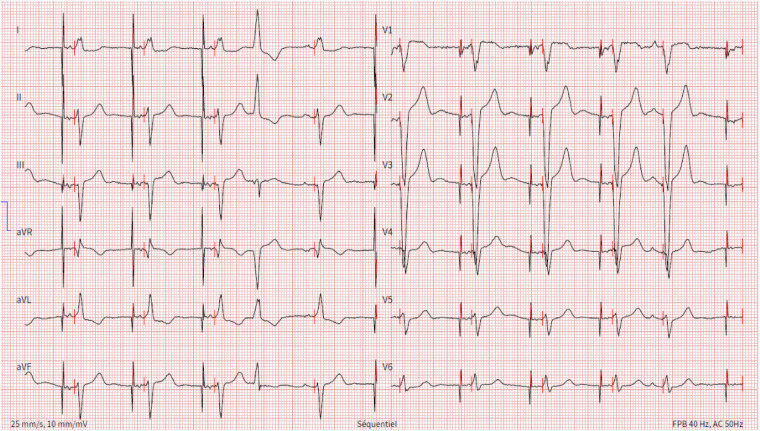
Electrocardiogram showing unipolar atrial stimulation by the transvenous pacemaker followed by a ventricular spike generated by the leadless pacemaker that leads to ventricular capture with a fixed atrioventricular delay, resulting in atrioventricular synchrony. One ventricular and one atrial ectopic beat are also present.

**Figure 4:**
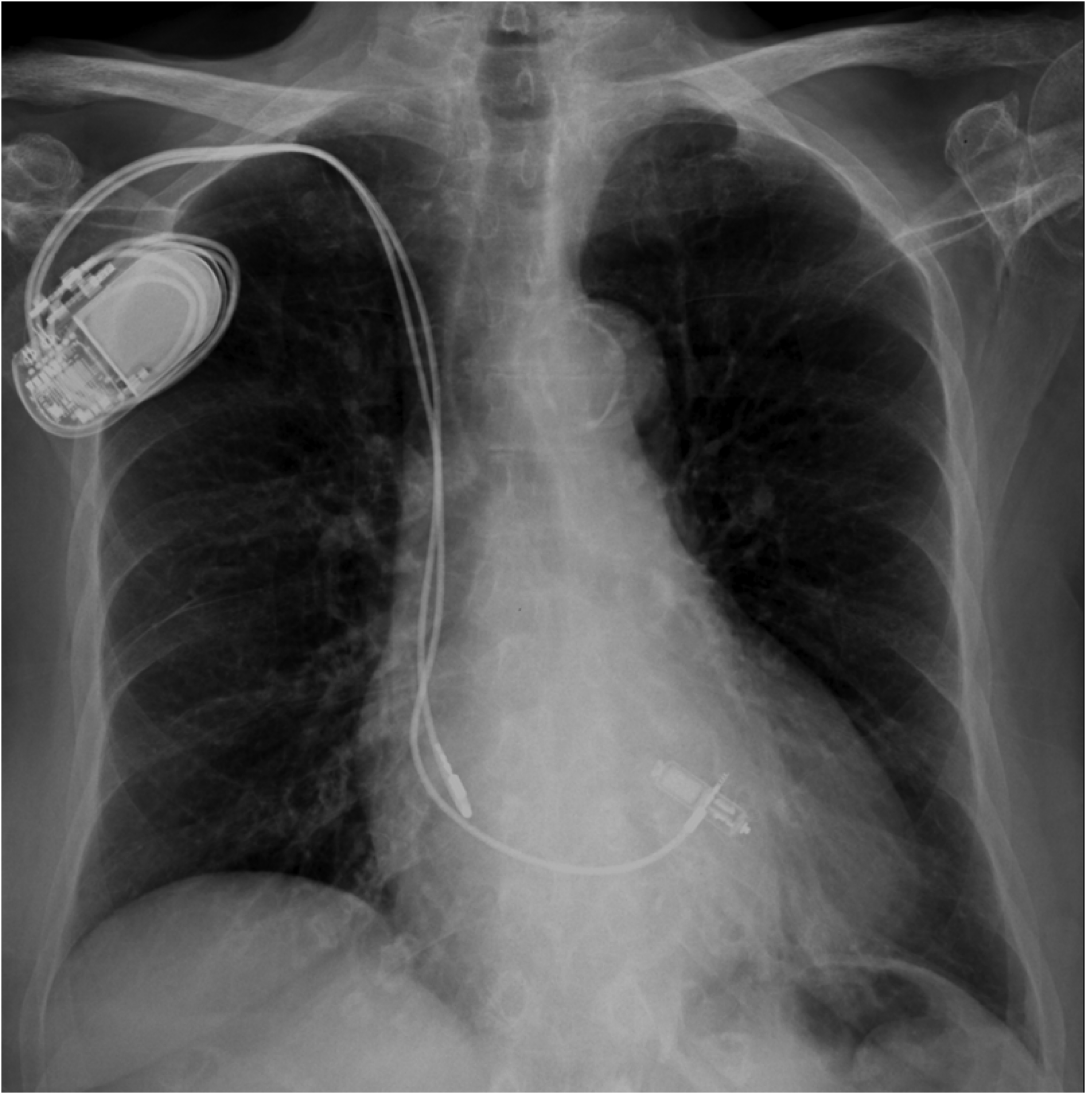
Post-procedural chest x-Ray showing a dual chamber transvenous pacemaker and a MICRA™ AV leadless pacemaker implanted at the interventricular septum.

**Figure 5:**
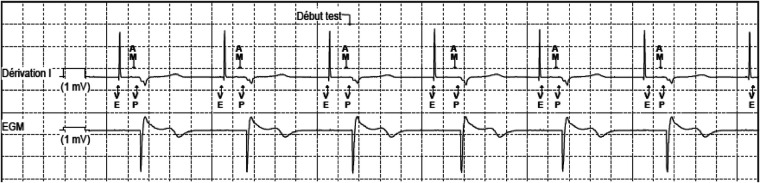
MICRA™ AV window. Lead DI shows a sharp and high-amplitude signal that corresponds to the atrial pacing stimulus. An atrial mechanical event is sensed during the A3 (passive ventricular filling phase)/A4 (active ventricular filling) window, marked as AM on the stip. After the predefined atrioventricular delay, the pacing stimulus is delivered (VP). The amplitude of the recorded atrial signal has been automatically decreased to adapt to the sensing of the high amplitude far-field atrial pacing stimulus. Nevertheless, the atrial activity is well recognized by the accelerometer and the resulting atrioventricular synchrony is effective. VE, defines the end of the A3 window; AM, presumed atrial mechanical contraction (A4 signal/A-wave); VP, ventricular pacing.

As the risk of CIEDs-related infection was high, with a calculated PADIT score at 7, representing a subsequent rate of hospitalization for device infection of 2.82% at one year, the patient was implanted with a leadless PM capable of maintaining AV synchrony in VDD mode (MICRA™ AV model MC1AVR1, Medtronic, MN, USA). The device was implanted at the level of the lower interventricular septum, as shown in Figure 2, since the mid-septum was difficult to access. During the implantation procedure, while performing the leadless stimulation threshold testing, no atrial retrograde conduction was observed. The transvenous PM was programmed to an AAI-R mode. With these settings, the transvenous PM was responsible for rate-responsive atrial stimulation, while the leadless PM allowed for atrial triggered ventricular stimulation (Figure 3). At day one after implantation, a chest x-ray showed no signs of displacement (Figure 4) and the leadless PM interrogation revealed satisfying parameters: stimulation threshold of 0,25 volts at a pulse width of 0,24 ms and an impedance 870 ohms. The patient was discharged one day after implantation. During follow-up, the electrocardiogram and device interrogation revealed stable stimulation parameters with an AV synchrony rate of 88% at one month (Figure 5) and 85% at six months (Table 1). An AAI-R mode setting was maintained for the transvenous PM. The patient had no perioperative or short-terms complications.

## 3. Timeline

**Table 1:** Timeline.

Time point	Event
15/11/2014	Transvenous dual chamber PM implantation
16/11/2014	Early ventricular lead displacement. Ventricular lead repositioning.
12/01/2023	Admission at the Emergency Department for syncope due to ventricular loss of capture.
13/01/2023	MICRA™ AV implantation
14/01/2023	Chest x-Ray and device interrogation. Hospital discharge.
21/02/2023	Device interrogation; AV synchrony rate: 88%.
16/06/2023	Device interrogation; AV synchrony rate: 85%

## 4. Discussion

Infection is one of the most threatening CIEDs complications, significantly increasing morbidity, mortality and healthcare costs (3, 4). Infection rates are known to be higher with device replacement procedures (5). Among others risk factors, early reintervention is also associated with an increased risk of infection (OR15.04; 95% CI, 6.7 to 33.73) (2). Renal insufficiency, even when moderate (GFR ≤60 cc/min/1.73 m^2^), dramatically increases the risk of infection (6). Recent technological advances introduced the use of leadless PMs. Their main advantage is the absence of a subcutaneous pocket and transvenous leads, resulting in a very low rate of device-related infections (7). Originally, these devices where only able to sense and stimulate the right ventricle (i.e., VVI mode) (8). The potential benefits of atrial pacing in patients with sinus node dysfunction, as well as the recognized benefits of AV synchrony mandated further development of leadless PMs. Atrioventricular synchrony decreases the incidence of pacemaker syndrome, improves stroke volume and positively influences functional status and quality of life of patients with AV block (9–11). Recently, the second-generation of the most widely used leadless PM, the MICRA^TM^ AV model MC1AVR1 (Medtronic, MN, USA) was introduced to widen the spectrum of patients who qualify for leadless pacing. This device provides contactless atrial sensing and allows for AV synchronous ventricular stimulation in VDD mode. Atrial sensing relies on mechanical detection of the atrial contraction via an integrated accelerometer-based sensor. Atrioventricular synchrony in early short-term feasibility studies ranged from 60%–90%, even though it was heavily dependent on patient's activity level and intrinsic AV conduction (12, 13). A recent study showed that a high AV synchrony rate can be predicted by an E/A ratio < 0.94 and a low sinus rate variability at rest (standard deviation of successive differences of P-P intervals < 5 bpm) (14).

The patient presented in this case was a fragile 90-year-old woman, initially implanted with a dual chamber PM for sinus node dysfunction, who subsequently developed a complete AV block. She had several risk factors for device related infection: a history of early device reintervention, a ventricular lead dysfunction and renal impairment. We therefore decided to implant a leadless PM programmed in a VDD mode to minimize the risk of device infection while, at the same time, preserving AV synchrony. The AV synchrony rate was satisfying at 88% at one month follow-sup, and 85% at 6 months The relative stable atrial stimulation by the transvenous PM in AAI-R mode in a patient with a low level of physical activity seems to account for the observed high level of AV synchrony.

## Data Availability

The original contributions presented in the study are included in the article/Supplementary Material, further inquiries can be directed to the corresponding author.

## References

[1] Sohail MR Henrikson CA Braid-Forbes MJ Forbes KF Lerner DJ. Mortality and cost associated with cardiovascular implantable electronic device infections. Arch Intern Med. (2011) 171(20):1821–8. 10.1001/archinternmed.2011.441 21911623

[2] Klug D Balde M Pavin D Hidden-Lucet F Clementy J Sadoul N Risk factors related to infections of implanted pacemakers and cardioverter-defibrillators. Circulation. (2007) 116(12):1349–55. 10.1161/CIRCULATIONAHA.106.678664 17724263

[3] Clémenty N Carion PL de Léotoing L Lamarsalle L Wilquin-Bequet F Brown B Infections and associated costs following cardiovascular implantable electronic device implantations: a nationwide cohort study. Europace. (2018) 20(12):1974–80. 10.1093/europace/eux387 29672690

[4] Habib G Lancellotti P Antunes MJ Bongiorni MG Casalta JP Del Zotti F 2015 ESC guidelines for the management of infective endocarditis: the task force for the management of infective endocarditis of the European Society of Cardiology (ESC). Endorsed by: European Association for Cardio-Thoracic Surgery (EACTS), the European Association of Nuclear Medicine (EANM). Eur Heart J. (2015) 36(44):3075–128. 10.1093/eurheartj/ehv319 26320109

[5] Poole JE Gleva MJ Mela T Chung MK Uslan DZ Borge R Complication rates associated with pacemaker or implantable cardioverter-defibrillator generator replacements and upgrade procedures: results from the REPLACE registry. Circulation. (2010) 122(16):1553–61. 10.1161/CIRCULATIONAHA.110.976076 20921437

[6] Bloom H Heeke B Leon A Mera F Delurgio D Beshai J Renal insufficiency and the risk of infection from pacemaker or defibrillator surgery. Pacing Clin Electrophysiol. (2006) 29(2):142–5. 10.1111/j.1540-8159.2006.00307.x 16492298

[7] El-Chami MF Bonner M Holbrook R Stromberg K Mayotte J Molan A Leadless pacemakers reduce risk of device-related infection: review of the potential mechanisms. Heart Rhythm. (2020) 17(8):1393–7. 10.1016/j.hrthm.2020.03.019 32247833

[8] Boersma LV El-Chami M Steinwender C Lambiase P Murgatroyd F Mela T Practical considerations, indications, and future perspectives for leadless and extravascular cardiac implantable electronic devices: a position paper by EHRA/HRS/LAHRS/APHRS. Europace. (2022) 24(10):1691–708. 10.1093/europace/euac066 35912932 PMC13453153

[9] Lamas GA Ellenbogen KA. Evidence base for pacemaker mode selection. Circulation. (2004) 109(4):443–51. 10.1161/01.CIR.0000115642.05037.0E 14757681

[10] Lamas GA Orav EJ Stambler BS Ellenbogen KA Sgarbossa EB Huang SK Quality of life and clinical outcomes in elderly patients treated with ventricular pacing as compared with dual-chamber pacing. Pacemaker Selection in the Elderly Investigators. N Engl J Med. (1998) 338(16):1097–104. 10.1056/NEJM199804163381602 9545357

[11] Nielsen JC Andersen HR Thomsen PE Thuesen L Mortensen PT Vesterlund T Heart failure and echocardiographic changes during long-term follow-up of patients with sick sinus syndrome randomized to single-chamber atrial or ventricular pacing. Circulation. (1998) 97(10):987–95. 10.1161/01.CIR.97.10.987 9529267

[12] Chinitz L Ritter P Khelae SK Iacopino S Garweg C Grazia-Bongiorni M Accelerometer-based atrioventricular synchronous pacing with a ventricular leadless pacemaker: results from the Micra atrioventricular feasibility studies. Heart Rhythm. (2018) 15(9):1363–71. 10.1016/j.hrthm.2018.05.004 29758405

[13] Steinwender C Khelae SK Garweg C Chan JYS Ritter P Johansen JB Atrioventricular synchronous pacing using a leadless ventricular pacemaker: results from the MARVEL 2 study. JACC Clin Electrophysiol. (2020) 6(1):94–106. 10.1016/j.jacep.2019.10.017 31709982

[14] Garweg C Khelae SK Steinwender C Chan JYS Ritter P Johansen JB Predictors of atrial mechanical sensing and atrioventricular synchrony with a leadless ventricular pacemaker: results from the MARVEL 2 study. Heart Rhythm. (2020) 17(12):2037–45. 10.1016/j.hrthm.2020.07.024 32717315

